# Information Fusion in Autonomous Vehicle Using Artificial Neural Group Key Synchronization

**DOI:** 10.3390/s22041652

**Published:** 2022-02-20

**Authors:** Mohammad Zubair Khan, Arindam Sarkar, Hamza Ghandorh, Maha Driss, Wadii Boulila

**Affiliations:** 1 Department of Computer Science and Information, Taibah University, Medina 42353, Saudi Arabia; 2 Department of Computer Science and Electronics, Ramakrishna Mission Vidyamandira, Belur Math, Howrah 711202, West Bengal, India; 3 College of Computer Science and Engineering, Taibah University, Medina 42353, Saudi Arabia; hghandorh@taibahu.edu.sa; 4 Security Engineering Lab, CCIS, Prince Sultan University, Riyadh 12435, Saudi Arabia; mdriss@psu.edu.sa; 5 Robotics and Internet-of-Things Laboratory, Prince Sultan University, Riyadh 12435, Saudi Arabia; wboulila@psu.edu.sa

**Keywords:** vehicle-to-everything (V2X), mutual intelligent transportation (MIT), general purpose graphic processing unit (GPGPU), neural synchronization

## Abstract

Information fusion in automated vehicle for various datatypes emanating from many resources is the foundation for making choices in intelligent transportation autonomous cars. To facilitate data sharing, a variety of communication methods have been integrated to build a diverse V2X infrastructure. However, information fusion security frameworks are currently intended for specific application instances, that are insufficient to fulfill the overall requirements of Mutual Intelligent Transportation Systems (MITS). In this work, a data fusion security infrastructure has been developed with varying degrees of trust. Furthermore, in the V2X heterogeneous networks, this paper offers an efficient and effective information fusion security mechanism for multiple sources and multiple type data sharing. An area-based PKI architecture with speed provided by a Graphic Processing Unit (GPU) is given in especially for artificial neural synchronization-based quick group key exchange. A parametric test is performed to ensure that the proposed data fusion trust solution meets the stringent delay requirements of V2X systems. The efficiency of the suggested method is tested, and the results show that it surpasses similar strategies already in use.

0pt plus 1fil0pt plus 1fil

## 1. Introduction

Mutual Intelligent Transport Systems (MITS) are transportation systems in which two or more ITS sub-systems (personal, car, roadside, and centralized) facilitate and deliver an ITS solution with higher quality and service level than if only one of the ITS sub-systems worked together. MITS will employ sophisticated ad hoc short-range communication technologies (such as ETSI ITS G5) as well as complementary wide-area communication technologies (such as 3G, 4G, and 5G) to allow road vehicles to communicate with other vehicles, traffic signals, roadside infrastructural facilities, and other road users. Vehicle-to-vehicle (V2V), vehicle-to-infrastructure (V2I), and vehicle-to-person (V2P) interactions are all terms used to describe cooperative V2X systems.

Nowadays, data amalgamation in MITS has shown to be extremely effective in making the transport systems easier and safer. By minimizing traffic jams, offering smart transport techniques, drastically decreasing the frequency of road accidents, and eventually achieving autonomous vehicles, the data-based technologies implemented in MITS promise to profoundly transform a person’s driving experiences [1,2,3].

**Figure 1:**
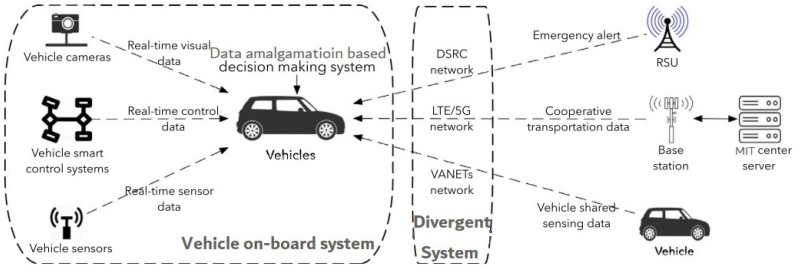
Information gathering and amalgamated system for intelligent decision.

Data may be gathered and amalgamated on many MITS modules due to the advancement of software and hardware. Due to transportation-based communications networks, people are living in the age of big data. Vehicles are equipped with smart modules for data gatherings, such as vehicular cams, detectors, and advanced control technologies, as depicted in Figure 1. As an outcome, the variation and quantity of data acquired by these systems for both physical and cyber settings is quickly rising [4]. However, at the other side, data blending and analyzing techniques, particularly Artificially Intelligent (AI) techniques, have improved to satisfy the actual data amalgamation requirements of MITS channels.

Wi-Fi connection between vehicles and other devices in MITS channels is another essential and basic technique for data blending in MITS infrastructure. IEEE 802.11p [5] and cellular-based [6] Wi-Fi transmission technologies are the two primary kinds of Wi-Fi transmission technologies for V2X data transmission. There are DSRC (Dedicated Short-Range Communications) guidelines in the United States [5] and Smart Transport System (STS)-G5 guidelines in Europe [5] for IEEE 802.11p standard-based transmission technologies. IEEE 802.11p can fulfill the most rigorous performance requirements for most Vehicle-to-Everything (V2X) applications. It is also possible to employ mobile connectivity technologies, such as LTE and 5G, to create uninterrupted and reliable network linkages, such as the connection between vehicles and remote servers. Figure 1 shows an example of how several transmission techniques are implemented in a divergent system for information sharing and information combination-based intelligent decision-making.

V2X transmission has been developed and categorized as per the sender and receiver [7] for exchanging data in this divergent data transmission system: Vehicle-to-Infrastructure (V2I), Vehicle-tof-Vehicle (V2V), Vehicle-to-Network (V2N), and Vehicle-to-Pedestrian (V2P). In essence, such concept is being used to further categorize the gathered information at the application level in order to implement information fusion-based intelligent decision-making processes on a single vehicle. V2I and V2V are by far the potential information transfer methods for upcoming MITS systems among the four forms of V2X data transmission [7]. Most efficient smart decision may be derived via data amalgamation and analysis based on information gathered from the smart detectors (cameras, sensor systems, radars, and controllers) and obtained from divergent V2X network systems. These smart choices made on every vehicle can subsequently be transmitted to other parts of the MITS network for traffic system improvement. Mutual information fusion may offer drivers a clearer view of a junction and assist them to identify other vehicles or walkers that they might otherwise miss owing to complicated driving situations such as barriers, disturbance, or adverse weather. Data amalgamation-based technologies on V2X divergent networks will aid in the implementation of autonomous vehicles on highways in the coming time.

Data interchange between vehicles and other elements in MITS systems has become more difficult with the advent of data amalgamation in the V2X data transmission systems, as multiple data types are exchanged over divergent networks. Many of these type of data are crucial to transportation and security systems, particularly in the context of driver-less driving. As a result, one key question is whether the acquired data via V2X systems can be trustworthy, particularly when it is utilized for security-related decision-making. An intruder may, for instance, corrupt the sending system and broadcast fraudulent or incorrect data to corrupt the entire MITS system. Furthermore, data carried via the V2X system may even be tampered with by an intruder to deceive vehicles, perhaps resulting in road accidents. An intruder can obtain the monthly broadcasts Cooperative Awareness Messages (CAM) [8] including the vehicle’s location, speed, and certain confidential material, allowing for eavesdropping and unlawful monitoring.

To create the security framework for data transferred in the V2X systems, solutions such as security and data privacy protection systems are developed. In related research, a Public Key Infrastructure (PKI) [9] has been used to share ephemeral keys to ensure data transfer safety and confidentiality in V2X platforms. Security methods such as key distribution as well as other safety computations such as procedures that provide information source authenticity, information content consistency, and information encoding, however, add significant delay. As security-related apps demand ultra-low delay, V2X is not projected to have a delay tolerance (e.g., cautions before a crash a total delay of less than 50 ms is usually required [6]). As a result, establishing an effective data amalgamation trust mechanism for data sharing in V2X divergent systems is a critical first step in advancing data amalgamation in MITS. Because communication apps rely on cluster communications, group-based safety features are crucial. The authenticity and security of the information that flows among others should be assured, which can be accomplished by Group key Agreement (GKA). GKA can accommodate both dynamic and static groups. GKA approaches are categorized into three parts: dispersed, contributing, and centralized.

For systems that require several users to communicate across open networks, security is a major concern. Among the most important components of protecting group communication is group key organization. The majority of group key distribution study is motivated by the original Diffie–Hellman (DH) key agreement technique’s main premise. This notion that there are costly exponentially procedures to be performed out will be the main disadvantage of generalized DH for multi-party. Transmission rounds and computing costs are two elements that impact the capacity of a GKA method. The idea of neuronal encryption, which is built on Tree Parity Machine (TPM) cooperative training, is expanded to processes in this article. Transmission loops and computing costs are two elements that impact the capacity of a GKA method. The group key was created for a participatory group key establishment mechanism which verifies participants and enables individuals to construct their key pair. It was also discovered that neural network-based GKA methods accomplish key validity and key concealment. While one method relies on an even more accurate link among quantity volume and price, another method concentrates on quantity demand and commitment to innovation.

The most important challenges are:

1. Despite the different ideas of data amalgamation security in previous research, a universal data amalgamation security framework is still required.
2. Minimization of delay caused by key distribution having a PKI framework in the current method for offering security functionalities for required data authentication in V2X data transmission is necessary.
3. As, on integrated systems in the vehicle, computations relating to security are always computationally intensive due to data source verification and data integrity checking to obtain the third degree of confidence for more data amalgamation, optimization of delay in the calculation is having a higher priority.
4. The establishment of a positional key agreement method that may may considerably minimize the delay caused by current key distributions is required.
5. A speedy calculation computing solution is required that fully utilizes the vehicles’ onboard GPUs for safety computation.
6. To improve confidentiality and effectiveness, a method for coordinating the neural group key swap-over process should be developed.

The main contribution of this paper is to build a data fusion security architecture with variable levels of trust. This research also provides an efficient and effective information fusion security solution for various sources and multiple types of data sharing in V2X heterogeneous networks. For artificial neural synchronization-based rapid group key exchange, an area-based PKI infrastructure with speed enabled by a Graphics Processing Unit (GPU) is offered. To confirm that the suggested data fusion trust solution fulfill the rigorous delay requirements of V2X systems, a parametric test is performed. This article discusses the benefits of implementing an area-based key pre-distribution scheme. First, by lowering request volumes, the area-based key distribution strategy can minimize the mean delay. The mean delay can be reduced with a high accuracy prediction rate.

Secondly, key distribution is a sort of information exchange procedure that includes the transmission of data as well as the essential security computations, whereas key requests/responses are a data type that is communicated in V2X heterogeneous networks.

Third, GPU speeding reduces the time spent on security-related computation operations. Therefore, information exchange delay in V2X networks involving cars and RSUs is significantly lowered for important request/response.

The section provides an overview of the paper’s different significant contributions:

1. This paper offers four levels of trust for the data amalgamation trust system which contain (i) there are no safety features, (ii) verified source of data, (iii) the authenticity, as well as the integrity of the data source has been verified, (iv) the data source has been confirmed, the data integrity has been checked, and the data content has been encoded.
2. To achieve the third degree of belief for information fusion in V2X systems a key exchange system as well as safety computation activities has been implemented so that the extra delay of implementing security methods must be taken into account, as the delay criteria in this data amalgamation process is stringent because safety is a top priority.
3. The delay has been decreased by minimizing key request updates with a position-based key dispersal network.
4. As per this research, the key exchange process must be developed by synchronizing a group of Triple Layer Tree Parity Machines (TLTPM). Rather than synchronizing individual TLTPM, the cluster members can utilize the identical key by cooperating among some of the selected skippers TLTPMs in logarithmic time.
5. The secret key is created by exchanging very few parameters across an unprotected link while both sides use the neuronal coordination procedure.
6. The proposed technique’s coordination duration for various learning rules are substantially fewer than the existing techniques.
7. The key swap over strategies described by [10,11,12,13,14] were investigated in the present study. This research focused on their weaknesses as well. To overcome the relevant problems, this article gives a TLTPM coordinating key agreement technique that results in a secret key with a flexible size.

Section 2 delves into related work. Section 3 contains the suggested approach. Section 4 deals with results and discussions. A conclusion and recommendation for future are presented in Section 5.

## 2. Related Work

The first degree of trust is used in some models [15], but it appears to be ineffective. PKI was used in certain models to share keys for identity verification, achieving the second degree of trust. The third degree of trust should be used for routine V2X data transfer, with fourth degree of trust accessible as a backup strategy for critical data transfer.

**Figure 2:**
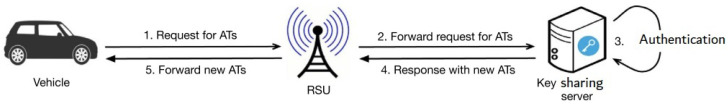
PKI-based key shairing procedure in [9].

The shared keys for vehicle transmissions in this network are temporary keys known as Activation Tokens (ATs). ATs are distributed once every small length of time (e.g., 10 min). As depicted in Figure 2, the procedure begins with a request from one vehicle to update ATs to RSUs, which is then forwarded to a distant key exchange platform, which validates the identification and replies. The vehicles and RSUs remain inactive throughout this period until the key sharing server responds. If the vehicle’s request is genuine, RSUs will transfer fresh ATs to the vehicle for use in the following time frame. In a different application scenario described in [9], the RSU verifies the digital certificate of the updated AT queries before forwarding them if they are legitimate. On the RSU side, this will undoubtedly result in longer delays. Without taking into account the delay between RSUs and PKI servers, the overall delay for AT upgrading may be over 400 ms. Furthermore, this delay will occur every 10 min, potentially affecting V2X transmission, particularly security messages, which have a 50 ms time restriction.

**Figure 3:**
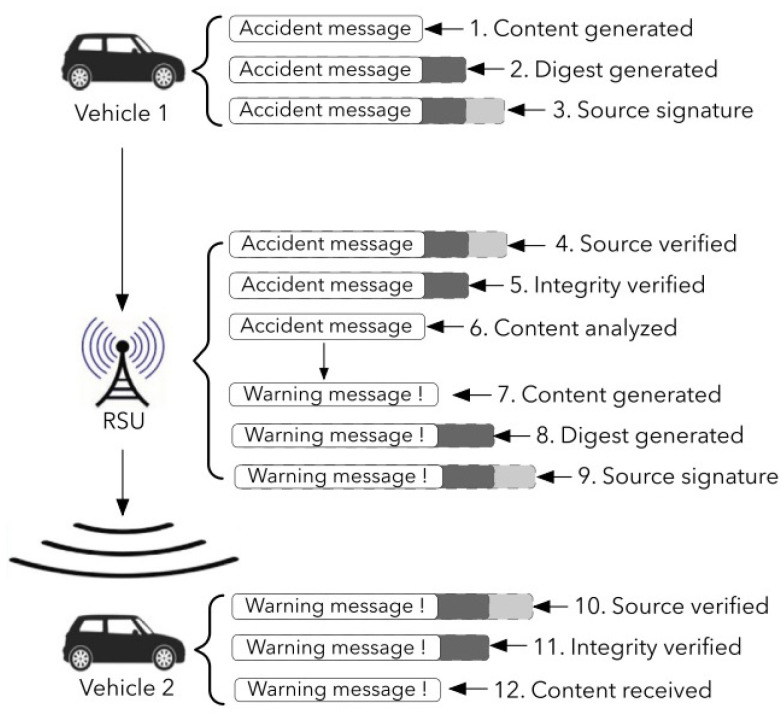
Exchange of information in urgent situation.

It is presumed that the V2X data transmission uses a public-key cryptosystem to validate V2X messages. The data of the V2X communication are not encoded in this case. Vehicle 1 would wish to send security information regarding an urgent situation, such as a vehicle breakdown, as depicted in Figure 3. This information will be created and structured in accordance with the V2X protocol, which includes both the payload and safety portions of the V2X information. A message-digest algorithm, such as the HASH, will also be used to create the specific pattern for consistency authentication in this V2X message. The given information will then be validated using a digital signature. When a neighboring RSU gets V2X information, it will first verify the information source’s authenticity and information integrity before accessing the content of the information. After all safety-related parts have been validated, the information will be processed and warning information will be sent to all surrounding cars. The RSU would then have to execute the HASH and digital signature operations to transmit this warning information to adjacent vehicles. Before the information payload is processed, the given information and information integrity must be examined and validated by the cars in the vicinity.

Four hash functions and two ECDSA signatures and authentication procedures run on vehicles and the RSU, respectively, will create the delay caused by safety actions in this scenario. In [16], the researchers compare their research on real-world V2X systems. According to the results of [16], the ECDSA technique takes between 5.5 ms and 7.2 ms every transaction using a 224 and 256-bit key. For authentications, the ECDSA technique with 224-bit key takes 7.2 ms and the 256-bit key takes 9.4 ms, respectively.

The efficiency on ARM9 devices may be substantially slower, as the authors highlighted, with one authentication process taking 150 ms.

Many prior studies [17] on both the hardware and software sides have developed and improved Hash algorithms, notably SHA-2 functions. Yet, the energy and computation capacity of integrated CPUs in cars have always been restricted. Measurement of SHA-2 efficiency on ARM series CPUs may be used to make an estimate, and a single SHA2 computation will take less than 1 ms. According to [18], depending on the physical device and length of the input information, SHA-3 might be 2 to 3 times worse.

Ingemarsson et al. [19] present the very first multi-user key management system, which is a Diffie–Hellman modification based on the concept of symmetrical functions. Members are connected in a ring, with $USER_{j}$ accepting only information from $USER_{j-1}$ and transmitting to only $USER_{j+1}$. Member $USER_{j}$ is able to calculate the secret key after n−1 sessions. However, being closer to a silent attacker who can monitor the communication pathways between both the members, this method was found to be vulnerable. Steiner et al. [20] present modifications of the Ingemarsson guidelines, in which member $USER_{m}$ can calculate the secret key after completing the very first phase. In stage 2, the member $USER_{m}$ transmits messages to $USER_{m-1}$ then the end portion of the communication is used by $USER_{m-1}$ as the power of its arbitrary value $s_{m-1}$ to create a secret key. The text’s last m−2 portion is then sent to $USER_{m-2}$ In compared to the Ingemarsson et al. method, this technique requires lesser computing costs from the principal but takes twice as many sessions. No communications could be transmitted till the previous secrets signal has been acknowledged in this method. Steiner et al. proposed a different method to lower every user’s average computation. Additionally, separate rules for joining and leaving of members are included in [21]. A circular method is used in Burmester-Desmedt key (BD) [22]. Members are connected in a circle in this approach. The calculation of the secret key is conducted in two steps, every adjacent couple of principles $USER_{j}$ and $USER_{j+1}$ finishes the fundamental DH key interchange in the first step. Client $USER_{j}$, on the other hand, instead of calculating the secret key separately, calculates the proportion of its two secret key $Y_{j}$ with adjacent client. At step 2, every client transmits their $Y_{j}$ value, allowing any client to calculate the secret key. When users are organized in a logical line instead of a circle. A slight change in the secret key emerges, which now just comprises the adjacent exponent sets on the connection. The approach for calculating the secret key is therefore dependent on the user’s location on the line, and it is much more capable than [22], especially when there are a high number of users. Perrig [23] proposed a GKA technique in which users are arranged as leaves in a B-Tree. The advantage of Perrig’s technique over prior techniques is that the quantity of necessary synchronization steps is logarithmic in terms of the total number of users; however, the technique’s restriction is that it allows $2^{m}$ users. Kim et al. [24] have demonstrated that the tree-based key establishment technique is particularly useful when the group’s arrangement has to be changed without having to restart the entire technique. They had also demonstrated that by re-configuring the tree of keys, they could easily add and remove individuals. Asymmetric GKA was described by Wu et al. [25] and Zhang et al. [26], while Gu et al. [27] suggested a consolidated GKA technique to reduce encrypting time. In ad hoc networks, Konstantinou [28] proposed an ID-centred GKA mechanism including well constant round. Jarecki et al. [29] created a resilient GKA technique in 2011 that permits a group of people to launch a shared secret key despite network problems. Many group key establishment techniques that have been provided can be implemented in one phase, but they do not provide advance confidentiality [30]. Most of the DH generalizations presented to date need minimum of two stages to create the mutually agreed key. The Joux [31] technique is the GKA technique that may be executed in a single stage while still maintaining advance confidentiality; however, it can only function with three members. The researchers of [10] proposed the CVTPM, which uses complex integers for all control factors. It is unclear if the CVTPM can resist a majority threat because they only looked at the geometric threat. A VVTPM system has been proposed by Jeong et al. [12]. This approach, however, does not really provide an accurate synchronization assessment. Teodoro et al. [13] suggested putting TPM structure on an FPGA to conduct key exchange by mutual training of these machines. Alieve et al. [32] presented a safe, lightweight, and scalable group key distribution and message encryption framework to tackle the secrecy of vehicle-to-vehicle (V2V) broadcasting. Leveraging scalable rekeying algorithms, the described group key management approach can handle diverse circumstances such as a node entering or leaving the group. Han et al. [33] developed a LoRa-based physical key generation technique for protecting V2V/V2I interactions. The communication is based on the Long Range (LoRa) protocol, that may use the Received Signal Strength Indicator (RSSI) to produce secure keys over long distances. Liu et al. [34] introduced recent conceptual conclusions on linked fractional-order recurrent neural networks’ global synchronization. The synchronization delay is quite long, and thus is not an effective strategy for group synchronization. Karakaya et al. [35] presented a memristive chaotic circuit-based True random bit generator and its realization on an Embedded system. The genuine randomness of the produced random number is not tested in this article using the NIST test suite. Dolecki and Kozera [14] investigated the sync times achieved for network weights picked at random from either a homogeneous or a Gaussian distribution with varying standard deviations. The network’s synchronization time is investigated as a function of various numbers of inputs and distinct weights pertaining to intervals of diverse widths. It is possible to correlate networks with various weight intervals; the deviation of a Gaussian distribution is chosen based on this interval size, which is also a novel way for determining the distribution’s variables. Patidar et al. [36] proposed a chaotic logistic map-based pseudo random bit generator. The genuine randomness of the produced random number is not examined in this work utilizing the 15 NIST test suite. A chaotic PRBG system based on a non-stationary logistic map was developed by Liu et al. [37]. The researchers devise a dynamic approach to convert a non-random argument sequence into a random-like sequence. The changeable parameters cause the system’s phase space to be disrupted, allowing it to successfully withstand phase space rebuilding attempts.

In conclusion, the overall delay in Figure 3 generated only by safety-based computations may be approximated at 30–40 ms. Data communication delay, data processing delay, and data producing delay are all examples of delays that are overlooked. This implementation and hardware configuration appears to be insufficient for V2X data transmission networks to share security-based data (50 ms delay). As a result, safety-based functions will need to be accelerated as well.

## 3. Proposed Methodology

This paper proposes an area-based key sharing system for cars and RSUs in this part, with pre-estimated and previously shared key pair pools. The temporary keys are updated in this system depending on the positions of the vehicles rather than the time frame. Interim keys (ATs) are previously-shared keys that are distributed to vehicles and RSUs for a limited period (e.g., 24 h). When a vehicle moves to a new region, the interim keys are updated to reflect the new location. Updated key requests may be disregarded as long as the cars are in regions with previously shared keys. Keys will be updated during non-peak hours (e.g., midnight) for use in the upcoming time frame. The use of a smart route prediction system can aid in the assumptions of areas through which one vehicle may traverse to access additional key sharing.

The researchers of [10] proposed the CVTPM, which uses complex integers for all control factors. It is unclear if the CVTPM can resist a majority threat because they only looked at the geometric threat. A VVTPM system has been proposed by Jeong et al. [12]. This approach, however, does not really provide an accurate synchronization assessment. Teodoro et al. [13] suggested putting TPM structure on an FPGA to conduct key exchange by mutual training of these machines.

The proposed architecture’s original concept is to share keys depending on the location of a single car rather than predetermined time intervals. The key pairs used for V2X transmission are modified based on the position information of the vehicle.

**Figure 4:**
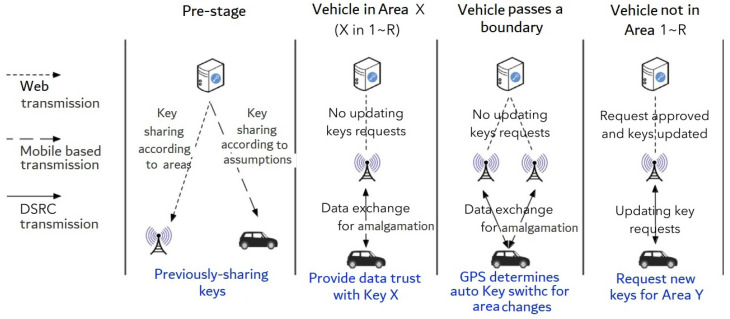
Proposed an area-based key sharing system for cars and RSUs.

Here’s an instance: Assuming the V2X transmission system is set up in a broad region with a single key sharing center, several RSUs, and a huge number of vehicles. To begin, this region is divided into *N* zones, each of which corresponds to *N* types of secret key stores. RSUs are classified into regions depending on their location. The RSUs are then assigned key sets that correspond to their region. After that, *R* sets of keys (key sets ranging from 1∼R) are previously-shared to one car, with proper identification confirmed. This part, seen in Figure 4, is known as pre-stage, and it is carried out at the start of each effective time frame (e.g., 24 h). If a vehicle remains in region *X* and *X* corresponds to 1∼R, the key sequences *X* can be used for V2X transmission. As a consequence, as soon as one car stays inside one region with previously-shared keys, no need to change keys inside a permissible time frame. When a vehicle reaches a zone without having received previously-shared keys, a request for related keys will be made to PKI servers via RSUs, just as the current V2X PKI network. As a result, by lowering the number of requests for key updates, the average key sharing delay may be decreased.

In the first stage of this network, the very first step is to share the keys. A cellular-based transmission network should be used since this phase necessitates data transmission between vehicles/RSUs and PKI servers. As previously said, one suitable time period is 24 h, and the previously-mentioned sharing step should occur at a non-rush hours, such as late at night. While the keys from the previous time session will still be in effect, the pre-stage in Figure 4 can be configured for one hour. RSUs will get fresh keys and relevant data via internet connections at this pre-stage. The majority of vehicles on the streets at present moment are unable to download new keys with previous-authentication using cellphone-based internet networks such as LTE or 5G connections. Meanwhile, there will still be automobiles on the roads that use the old keys to connect. Once the pre-stage is complete, the cars will rely on the currently requested keys as a backup mechanism until they can establish a robust link with the PKI server through mobile communication.

If a vehicle arrives in a region without previously shared keys, it will seek ATs as a complement to the key sharing mechanism. RSUs will send the request to the key sharing unit, and if authentication is satisfactory, new ATs will be returned, as shown in Figure 4. In this scenario, the delay is still present, exactly as it was in Figure 2. Still, with the proposed architecture, average requests for upgrading keys may be much decreased, resulting in a lower average delay for upgrading keys. The driving path prediction may be used to forecast the passing regions in the following time frame for single vehicle, which can help optimize this architecture.

When this design is first implemented, there is no way of knowing which key pools each vehicle should download. Assuming that the inbuilt computer of a vehicle has small memory space, requests for ATs related with unshared zones cannot be ignored.

The proposed key sharing method, must be utilized in collaboration with the present requests and responses-based key sharing system. The supplemental techniques are queries to PKI servers for key updates. If the zones related with previously-shared keys are *R*, and the entire areas one car actually crossed are *M*, then It may be deduced that if R⊆M, that refers to a single car, has keys for all of the passing areas, no requests for key updates will be made. If M⊆R, the car will require the old key upgrading mechanism as a backup for acquiring new ATs in zones where previously-shared keys are not available. As a result, while delay cannot be prevented in this situation, requests are still minimized. Apparently, if R={}, the vehicle will have to seek AT updates in all areas it has gone through. As a result, if the vehicle goes through one area in less than 10 min, there may be additional update requests.

To summarize, to decrease the number of requests for ATs, it is critical to predict each vehicle’s traveling areas and previously-shared appropriate keys. The potential traveling areas for one vehicle in the following span of time may then be estimated using a destination and path assumptions method.

Because this article is not about smart path or endpoint prediction, this paper presume a variable for the success ratio of path prediction in Section 5 and look at alternative efficiency assessment outcomes.

**Figure 5:**
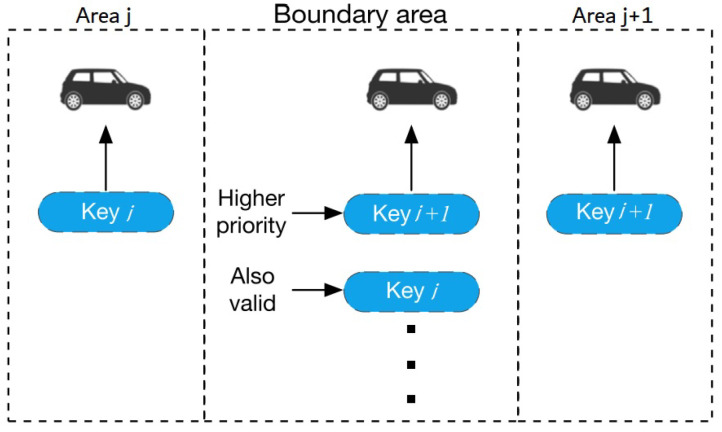
Key management in boardering area.

There will always be borders between neighboring regions since the design is dependent on geography. The key pair that is currently in use needs to be upgraded as per the GPS position as a vehicle crosses a boundary between two neighboring areas. There will be a period of time when connectivity is inaccessible owing to the changeover of the keys in this instance. In this architecture, this article built a border zone between two nearby regions in which both keys are permitted to utilize for automobiles and RSUs. After just a vehicle travels from region *j* to region j+1, there is indeed a region where both keys are available, as shown in Figure 5. The keys utilized for the next passing area will have precedence if the navigation system recognizes which area the car will be in next. If the vehicle is traveling from Area *j* to Area j+1, for example, Key j+1 will have greater priority than Key *j* as illustrated in Figure 5. Keys pertaining to preceding regions would have a smaller impact due to the lack of a defined priority based on the path prediction. Setting border zones has the primary purpose of maintaining connectivity by minimizing time delays caused by key flipping.

This design is based on the validation of one-time slot’s starting phase. After their genuine ID has been validated, the cars and RSUs will get the necessary shared key from the key sharing hub. During each time frame, a monitoring mechanism should be implemented to keep an eye on the abuse of the keys or other odd actions, such as sending misinformation to the MITS in one region. Any further exploitation of the ATs will result in the ID being re-verified, ensuring that an intruder does not have a genuine authenticate ID to acquire the keys in a single time period. As a result, after the attack has been observed, the assailants will be penalized in the following time period. To summarize, dangers persist, but the difficulty of attacking this security framework has increased, and attackers’ actual identities are easier to detect for subsequent operations.

Another advantage of this area-based PKI is the ability to update the keys more often. Because the keys for V2X communications must be changed to prevent unlawful monitoring, area-based PKI might allow several key pairs for a single block, allowing cars to change keys more often.

The definitions of the symbols used are depicted in Table 1.

A suitable replacement for number theory-based encryption methods is the transmission of secret keys across public networks utilizing mutual communication of neuronal networks. Two ANNs (Artificial Neural Network) were trained using the identical weight vector. The keys are the same weights acquired from the coordination. On a public network, this technique was utilized to create cryptographic keys. The ANNs will coordinate quicker due to the evident ideal matched weights created by neural coordination utilizing the identical input. These ideal input vectors not just to speed up the coordination process, but they also minimize the likelihood of adversaries. This technique of key creation is effective to the extent that if a new key is required for every communication, it may be created without storing any information. The network’s efficiency can be improved even more to acquire a session key on a transmission media by expanding the amount of nodes for either the input or concealed layer. The idea of neuronal encryption has been expanded to produce a group key in this article, in which the secret key consists of coordinated weights acquired via the ANN. The ANN technique for creating keys for two-party connectivity will now be expanded to produce keys for users connecting in groups. Every user will get his or her own ANN. ANNs begin with their own starting vectors and weight vectors, and coordination occurs when all of the ANNs in the cluster reach the identical weight vectors. The following subsections describe two forms of GKA-based on the primary setup. Section 3.1, Section 3.2, Section 3.3 are discussed synchronization using ring framework, synchronization using tree framework, and security assessment of proposed methodology.

### 3.1. Synchronization Using Ring Framework

**Figure 6:**
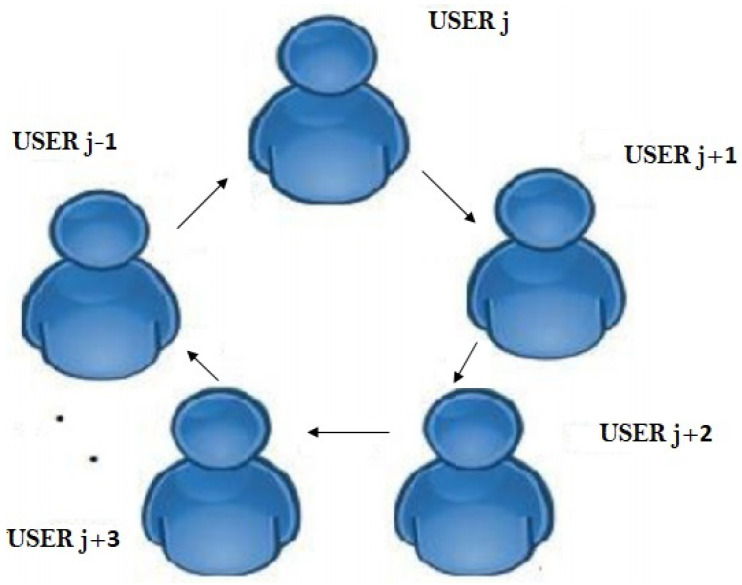
Ring framework.

As illustrated in Figure 6, the *m* users involved in the communication are organized in a ring, so that user $USER_{j}$ only takes messages from $USER_{j-1}$ and communicates with $USER_{j+1}$. The initial input vector and the weight range are distributed across the users. Here, flag *g* is used to indicate whether the output of TLTPMs of different user’s are same or not. If output of two TLTPMs are identical then flag set to 1. Otherwise, it is set to 0.

Phase One: Initialization:

1. Each member $USER_{j}$ will also have his or her own TLTPM, with starting weight.

2. Each member $USER_{j}$ would put the flag number g=1 and initialize his TLTPM with the shared input.

Phase two: Key Generation:

3. Until coordination, every principal $USER_{j}$ will carry out the procedures below.

(i) Calculate the values of the hidden neurons and the output neuron. Principal $USER_{j}$ now transmits its output and flag values to $USER_{j+1}$. Then, $USER_{j+1}$ compares its output to the received one. If both outputs are same then $g_{j}=1$ is kept. Otherwise, $USER_{j+1}$ reset the flag value to 0 and transfers it to $USER_{j+2}$.

(ii) If the flag parameter $g_{j}$ is initialized to zero at any time during the calculation, the subsequent users $USER_{k}\;\left(k=j+1,\;j+2\right)$ continue to propagate $g_{j}=0$ to the remaining principals in the ring until j≡kmodm. As a result, a newer flag value will be sent to the agreed-upon users.

(a) Go to step 2 if the flag g=0.

(b) If the flag g=1, each user $USER_{j}$ updates their weight vector according to the following learning rule (Equation (1)).

(1) $$\begin{array}{c} \begin{array}{c} \delta_{v,w}^{q+}=fun\left(\delta_{v,w}^{q}+\gamma_{v,w}^{q}\zeta \Phi \left(\xi_{v}^{q}\zeta \right)\Phi \left(\zeta^{G}\zeta^{D}\right)\right) \end{array} \end{array}$$

The Heaviside stage function is Φ

The keys are the same weights acquired from the coordination. The keys now can be utilized for the encoding/decoding. After agreeing on the neural key by the members of the group, it may be necessary for users to enter or quit the group in the ring structure. One method is to simply resurrect the method in order to generate a new key for the customized group.

### 3.2. Synchronization Using B-Tree Framework

**Figure 7:**
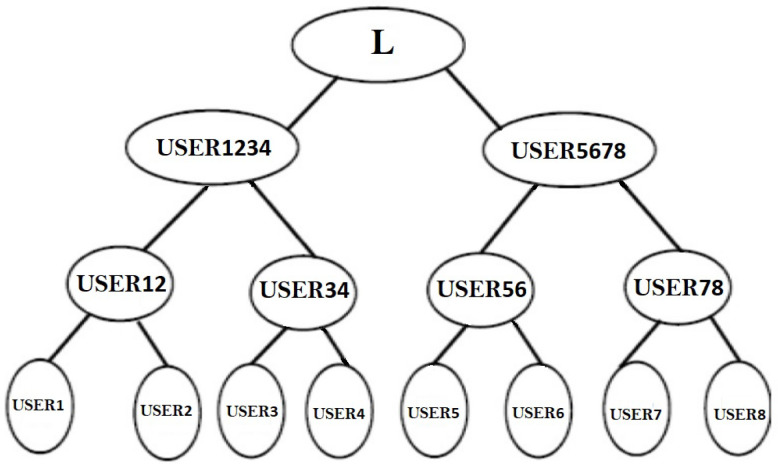
Tree framework considering m as even.

The terminal neurons of the B-Tree are represented by the main $USER_{j}\;\left(j=1\;to\;m\right)$ in the group in Figure 7. The neighboring principles $\left(USER_{j^{\prime }}\;USER_{j+1}\right)$ form a couple and begin with a shared starting input.

There will be m/2 pairings if the number of nodes is even. In Figure 7, we examine eight users who pair up as $\begin{array}{c} \left(USER\;1,\;USER\;2\right)\left(USER\;3,\;USER\;4\right)\\ \left(USER\;5,\;USER\;6\right)\left(USER\;7,\;USER\;8\right) \end{array}$.

Each pair forms a TLTPM to create the synchronized weights $\begin{array}{c} \left(USER\;12\right)\left(USER\;34\right)\\ \left(USER\;56\right)\left(USER\;78\right) \end{array}$. These synchronized weights are now the pair’s initial weights $\left(USER\;12\right)$. Similarly, $\left(USER\;34\right)\left(USER\;56\right)\left(USER\;78\right)$ have initial synchronized weights. In the following round, $\left(USER\;12,\;USER\;34\right)$ and $\left(USER\;56,\;USER\;78\right)$ create consecutive pairs for the TLTPM and produce keys $\left(USER\;1234,\;USER\;5678\right)$. The above mentioned merging and synchronization procedure is repeated until all users have merged to create a single group. Every member are linked to an identical weights that forms the neural group key.

**Figure 8:**
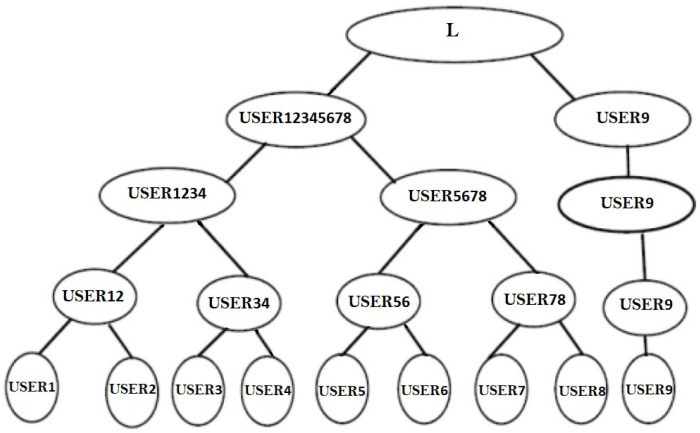
Tree framework considering m as odd.

For odd number of members, as illustrated in Figure 8, say $\left(2^{m}+1\right)$, the $2^{m}$ users will merge to an identical neural key as previously explained, while the final member will then coordinate the TLTPM with the TLTPM of USER12345678 to get a mutually agreed key. The amount of layers necessary for key exchange is determined by the amount of users in the cluster. That is, for $2^{m-1}<$*number of users*$\leq 2^{m}$ then *m* levels are required. For example, for m=3, we may have 5, 6, 7, or 8 members, and the amount of levels necessary for key creation stays the same for all of these users.

Algorithm 1 explains the whole TLTPM sync mechanism.
**Algorithm 1:** The whole TLTPM sync mechanism.**Input**: The random vector weight $D^{q}$ and the identical input $G^{q}$.**Output**: With the identical key pair, both sender and recipient have synchronized TLTPM.***Step 1:*** Assign the weight matrix $D^{q}$ to any random vector value. Where, $\delta_{v,w}^{q}\in \left\{-\lambda ,-\lambda +1,\;\cdots ,\;+\lambda \right\}$.Repeat steps 2 through 5 until you have achieved ideal synchronization.***Step 2:*** Every hidden node’s output is determined by a weight dependent on the current state of the inputs. The result of the first hidden layer is determined by Equation (2).(2)$$\begin{array}{c} \begin{array}{c} \kappa_{v}^{q}=\frac{1}{\sqrt{N}}\gamma_{v}^{q}.\delta_{v}^{q}=\frac{1}{\sqrt{N}}\sum_{w=1}^{N}\gamma_{v,w}^{q}\delta_{v,w}^{q} \end{array} \end{array}$$The result $\xi_{v}^{q}$ of the *v*-th concealed node is denoted by $signum(\kappa_{v})$ in Equation (3).(3)$$\begin{array}{c} \begin{array}{c} \xi_{v}^{q}=signum\left(\kappa_{v}\right) \end{array} \end{array}$$If $\kappa_{v}=0$ is true, $\xi_{v}^{q}$ is mapped to −1, then binary outcome is produced. $\xi_{v}^{q}$ is assigned to +1 if $\kappa_{v}>0$ is true, indicating that the concealed neuron is functioning. The concealed node is deactivated if the magnitude is $\xi_{v}^{q}\;=-1$. Equation (4) shows how to do this.(4)$$\begin{array}{c} \begin{array}{c} signum\left(\kappa_{v}\right)=\left\{\begin{array}{cc} -1 & if\;\kappa_{v}\leq 0\\ +1 & if\;\kappa_{v}>0 \end{array}\right. \end{array} \end{array}$$***Step 3:*** Calculate the final result of TLTPM. The ultimate result of TLTPM is calculated by multiplying the hidden neurons in the last layer. This is represented by ζ (Equation (5)).(5)$$\begin{array}{c} \begin{array}{c} \zeta =\overset{\tau 3}{\underset{v=1}{\prod \xi_{1}^{v}}}\;\overset{\tau 3}{\underset{v=1}{\prod \xi_{2}^{v}}}\;\vdots \;\overset{\tau 3}{\underset{v=1}{\prod \xi_{\varsigma }^{v}}} \end{array} \end{array}$$Equation (6) shows how the magnitude of ζ is represented.(6)$$\begin{array}{c} \begin{array}{c} \zeta =\left\{\begin{array}{cc} -1 & if\;\xi_{v}=-1,\;is\;odd\\ +1 & if\;\xi_{v}=-1,\;is\;even \end{array}\right. \end{array} \end{array}$$If TLTPM has one concealed neuron, $\zeta =\xi_{1}^{q}$. For $\;2^{\mu -1}$ distinct $\left(\xi_{1}^{q},\;\;{\xi_{2}^{q}}_{,\;\cdots ,\;}\xi_{\mu }^{q}\right)$ options, the ζvalue is the same.***Step 4:*** When the findings of two TLTPMs *G* and *D* agree, $\zeta^{G}=\zeta^{D}$ adjusts the weights using one of the following rules:Both TLTPMs will be learned from each other using the Hebbian learning method (Equation (7)).(7)$$\begin{array}{c} \begin{array}{c} \delta_{v,w}^{q+}=fun\left(\delta_{v,w}^{q}+\gamma_{v,w}^{q}\zeta \Phi \left(\xi_{v}^{q}\zeta \right)\Phi \left(\zeta^{G}\zeta^{D}\right)\right) \end{array} \end{array}$$If the weights for each TLTPM are the same, go to step 6.***Step 5:*** If the outcomes of the both TLTPMs differ, the weights cannot be altered, $\zeta^{G}\neq \zeta^{D}$. Go to step 2 now.***Step 6:*** As cryptographic keys, use these coordinated weights.

The procedure manages the joining and leaving of individuals from the group as below.

(i) Leaving from the grouping

**Figure 9:**
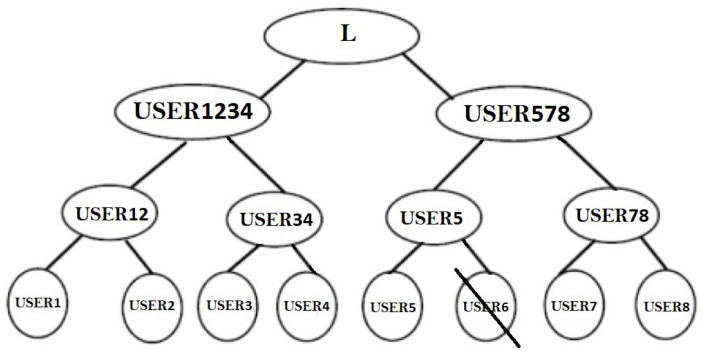
User 6 will leave the group.

Figure 9 depicts a situation in which a member USERj, let $\left(USER\;6\right)$, wishes to quit the group at level 2 after obtaining keys $\left(USER\;1234\right)$ and $\left(USER\;5678\right)$. It is now apparent that omitting $\left(USER\;6\right)$ will not result in a change to $\left(USER\;1234\right)$ obtained from the pair $\left(USER\;12\;USER\;34\right)$. As a result, these users do not need to execute their TLTPM once again to generate coordinated weight vector. The weights of $\left(USER\;56\right)$ impacts the $\left(USER\;5678\right)$, although $\left(USER\;78\right)$ can also keep their coordinated weight vector from level 1. That $\left(USER\;5\right)$ is a single node, its TLTPM will have initial weight vector and will coordinate with $\left(USER\;78\right)$ to obtain a pair of coordinated keys for $\left(USER\;578\right)$. After that, $\left(USER\;1234\right)$ and $\left(USER\;578\right)$ execute their separate TLTPMs to merge to the same weight vector, that becomes the group of principals’ neural key.

(ii) Joining to the group

**Figure 10:**
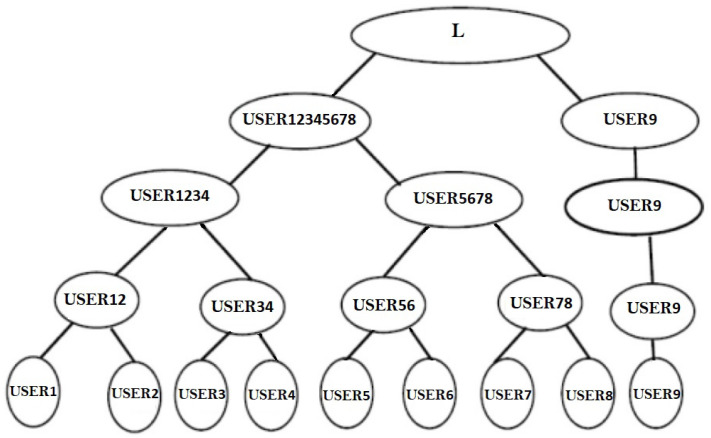
User 9 will join as a fresh node.

If any user $USER_{j}$ wishes to participate in the group, such as a fresh member$\left(USER\;9\right)$, $\left(USER\;9\right)$ get linked with the root of the tree, as illustrated in Figure 10. Previously, with the eight members, two levels are needed to construct the key, namely $\left(USER\;1,\;USER\;2\right)$$\left(USER\;3,\;USER\;4\right)\;\left(USER\;5,\;USER\;6\right)$ and $\left(USER\;7,\;USER\;8\right)$ instruct their networks in simultaneously at 0th, and then in 1st level $\left(USER\;12,\;USER\;34\right)\;$ and $\left(USER\;56,\;USER\;78\right)$ will instruct their networks for synchronization, and in level 2 $\left(USER\;1234,\;USER\;5678\right)$ will instruct their TLTPM’s and merge to an identical weight vector. Now, there are 9 participants in the group $\left(USER\;1,\;USER\;2,\;\;USER\;3,\;USER\;4,\;\cdots \cdots USER\;9\right)$. After level 2 $\left(USER\;1234,\;USER\;5678\right)\;$ instructed their network system and synced weight values, a new joining USER 9 in level 3 $\left(USER\;12345678,USER\;9\right)$ will develop as a pair and instruct their network systems for weight synchronization, as illustrated in Figure 10.

### 3.3. Assessment of Security

As safety computations are implemented, the delay in V2X transmission systems can also be raised, as demonstrated in Section 2. This method record all safety computations depending on the degree of confidence specified in this part. After that, a GPU-based architecture will be shown, with its efficiency compared to conventional systems.

Public-key encryption is used in existing V2X data transmission to verify over internet transmissions. Despite various variations between the US stack (IEEE 1609) and the EU stack (ETSI ITS G5) [5], the techniques used regarding safety are almost comparable, as per [38]. This paper categorize safety computations based on the needs. There are three primary types of security features in V2X data transmission for the most common use cases: information source authenticity, information content consistency, and information encoding.

#### 3.3.1. Authentication of the Information Source

To avoid the problems caused by fraudulent and incorrect data from spoofing attacks, a verification system is employed to ensure that the information is transmitted from a genuine source. The most practical approach is to employ digital authentications for both the initiator and the recipient to sign and check. The ECC technique was chosen by the standards owing to the lower size of signs and keys, as per [39]. Signs are generated by using ECDSA using 256 or 224-bit keys [38].

#### 3.3.2. The Consistency of Information Content

The data content integrity is achieved by generating a hash, that is always sent with the data to identify any change at the recipient’s end. SHA-2 functions, which generally include SHA-256 and SHA-512 functions, are the most commonly used Hash functions. The TUAK method set [40], a more newly formed second method set for validation and key formation, will be used in V2X transmissions, according to the most current ESTI publication.

#### 3.3.3. Encoding of Information

Encrypting every information sent is the best way to totally avoid snooping and the leakage of confidential data in V2X transmissions. Key sharing techniques are often used in most existing data transmission to provide verification between two transmission participants. The contents of the sent communication are then protected using symmetric encryption methods. In most data transmission techniques, however, encrypting all transactions is always computationally costly. V2X data transmission networks, as mentioned in Section 1, have limited tolerance for processing overhead. Encoding will be employed as a supplemental technique for transferring confidential data, which is a more feasible architecture.

#### 3.3.4. GPU Computing-Based Acceleration

In reality, there are additional acceleration techniques for ECDSA or the Hash function in V2X data transmission networks to achieve higher efficiency. According to [38], the specialized hardware accelerator can do 1500 and 2000 ECDSA signature and validation calculations per second, respectively. The author of [41] implements ECDSA on an Intel i7 CPU, at a speed of less than 0.1 ms for a single signing or validation process. However, because of performance of these computations is greatly dependent on hardware configuration, it is appropriate to develop a viable solution based on reasonable hardware estimates. For example, equipping each vehicle with a specialized high-performance security calculator is expensive. Or, for architectural or power usage reasons, the Intel i7 CPU is unlikely to be used in cars. The architecture should take into account the hardware resources that are available on today’s vehicles. One viable option is to do the computation operations on System on Chip (SoC) platforms, which are often found in embedded systems such as smartphones. As a result, SoC designs are already being used in today’s automobiles: Vehicles using Nvidia Drive PX (really Nvidia Tegra X1 [42]) systems were used to speed up AI algorithms [43].

**Figure 11:**
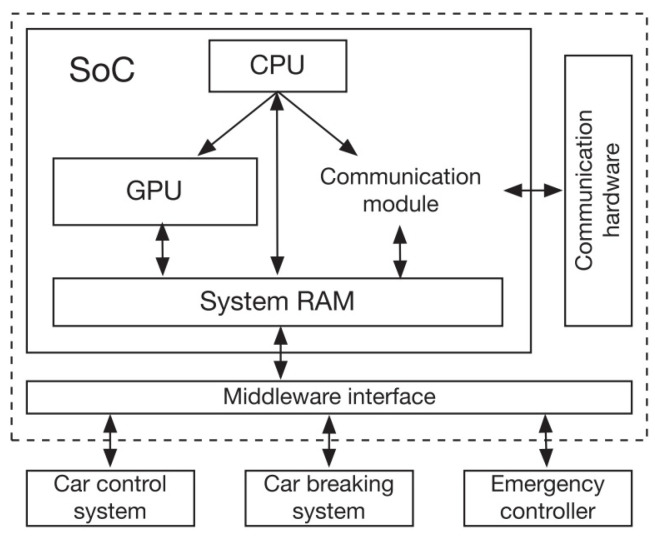
Mmimized SoC structure.

The Nvidia Tegra X1 is a conventional SoC design that includes a GPU, a visual encoder/decoder, DSP, and dashboard monitor, as well as a single part of systems RAM and a CPU that acts as control system and coordinators. As illustrated in Figure 11, this article construct the solution for V2X transmission systems using a minimized SoC structure with only the essential parts. The CPU is a computation core that also serves as a controller for other parts such as the GPU and communication modules. Information in the system RAM can be calculated by GPU, that can also be controlled by the CPU and units for communication. Small computation processes can then be assigned to various units for numerous complicated processes for overlapping the processing period for improved execution.

#### 3.3.5. ECDSA and Hash Computation on GPU

Nvidia has a number of various driving platforms that are ready to be installed in automobiles. The GPU on Nvidia SoC systems can be leveraged to speed safety computations, according to a study [44]. Singla et al. [44] provides an ECDSA benchmark on both GPU and CPU units for the Nvidia Tegra X1 system. In offline mode, the Tegra CPU can do one signature procedure in around 10 milliseconds, while the Tegra GPU can speed up the process by three times. When it comes to validating information, the Tegra CPU can do one checking process in less than 1 millisecond, which is quicker than the GPU. The experiments in [45] demonstrated that now the Hash techniques SHA-512 and SHA3 sequence can be accelerated on the Tegra architecture and that the performance meets the delay criteria.

As a result, Tegra’s CPU deployment speed may only be sufficient for security apps. The Tegra SoC platform can speed up safety-related computation considerably quicker than the findings in [16] and will fulfill the delay requirements of security apps because of GPU acceleration. Furthermore, by allocating computing jobs for a large number of inputs utilizing such SoC architectures, this technique may achieve better speeds.

Future confidentiality, reverse confidentiality, and key autonomy are safety objectives for dynamic organizations described by Kim et al. [24]. Future confidentiality ensures that an attacker who has a collection of cluster keys will be unable to deduce any consecutive group key. To put it another way, when a principle quits the group, the key he left behind has no bearing on the architecture of succeeding group keys. During joining, a new key is generated, which ensures reverse confidentiality. In a ring procedure, the participants begin with an arbitrary input and synchronize to produce a newer key value. When a new principle joins a group using a tree structure-based protocol, reverse confidentiality is ensured by generating a newer key. The fresh group key will be produced when the principal joins the current node, as illustrated in Figure 7. We may argue that key independence is also ensured if any attacker who has a collection of cluster keys are unable to form any other cluster key since the session is fully reliant on the arbitrariness of the input. The number of test steps is also shown to be directly related to the weight range *M*, slowing the coordination procedure and increasing the risk of intrusion. However, it helps to accelerate the coordination procedure when combined with an increase in *M* and the amount of concealed neurons. An attack’s success rate eventually drops as a result of this. As a result, the current protocol is protected against attack, and it is also important to specify which people may be trusted to protect data. A confidential key decided upon by both participants may be utilized to produce a static length hash associated with an information for this type of integrity verification. Hash functions is used to generate a data integrity certificate that safeguards the message’s authenticity and is transmitted with the message to safeguard it from illegal access. The communications are not hidden from an unknown party, but their integrity is guaranteed.

## 4. Results and Discussions

The performance analysis for information amalgamation trust structure is provided in this section. A diverging V2X system has been simulated using the NS-3 simulator, which includes a short-range transmission infrastructure amongst cars and RSUs as well as web guidelines amongst RSUs and the key sharing hub. The mean delay owing to the key sharing is computed for a region key sharing dispersal structure with distant prophecy accuracy ratios.

This method demonstrates the benefits of adopting an area-based key previously-sharing scheme in this simulation. First, by lowering request quantities, the area-based key sharing system can minimize the average delay. The average delay can be reduced with a high accuracy prediction ratio.

**Figure 12:**
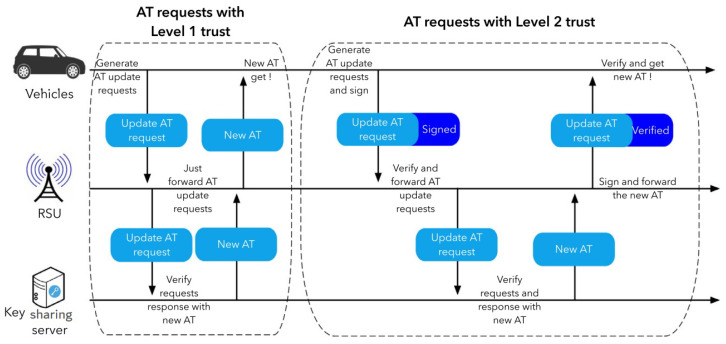
Current PKI solutions with two distinct degrees of confidence.

There are two types of key update processes matching two distinct trust levels in the present assessment for conventional key sharing system [9]. The two kinds of key update procedures are identified in Figure 12 as AT inquiries having tier 1 trust and AT inquiries having tier 2 trust. AT inquiries having tier 1 trust are unprotected, and RSUs are supposed to merely transmit anything from the vehicles or the key sharing servers. On cars and RSUs that confirmed the source of data in AT requests having tier 2 reliability, the request/response are all tested as well as evaluated.

When the cars reach an unexpected area, as mentioned in Section 3, this approach leverages current key sharing techniques as backup options. In this part, this method will use the area-based key sharing technique to predict the average delay of key sharing, with the current key sharing technique as a backup. $Time_{Vehi}$, $Time_{RSU}$, $Time_{Key\;Center}$, and $Time_{Netork}$ are the four elements of the overall key sharing delay for the one-time key update, as shown in Figure 12.

$Time_{Vehi}$ refers to the time it takes for the vehicle to generate the request message, sign it, get the response from the key distribution server, and verify the response.

$Time_{RSU}$ is the time it takes for the RSU ends to validate the key update request, forward it, get the response message from the key distribution server, and sign it.

The delay on the key distribution server is therefore included in $Time_{Key\;Center}$, which is simplified as verify and response.

$Time_{Netork}$ delay on a heterogeneous network comprises delay on the DSRC network between cars and RSUs, delay on the Internet between RSUs and key distribution servers, and random delay congestion on the link between RSUs and key distribution centers.

$Time_{Vehi}$ refers (Equation (8)) to the time it takes for the vehicle to produce the request information ($Time_{Gena}$), sign the request information ($Time_{Sign\;Vehi}$), get the reply from the key sharing server ($Time_{Get\;New\;AT}$), and check the reply ($Time_{Vfy\;Vehi}$).

(8) $$\begin{array}{c} \begin{array}{c} \begin{array}{c} Time_{Vehi}\;=\;Time_{Gena}\;+\;Time_{Sign\;Vehi}\;\\ +\;Time_{Get\;New\;AT}\;+\;Time_{Vfy\;Vehi} \end{array} \end{array} \end{array}$$

$Time_{RSU}$ is (Equation (9)) the delay for the RSU endpoints to check the key update request ($Time_{Vfy\;R}$), send it ($Time_{Frd}$), receive the reply information from the key sharing server ($Time_{Rcv}$), and sign it ($Time_{Sing\;R}$).

(9) $$\begin{array}{c} \begin{array}{c} Time_{RSU}\;=\;Time_{Vfy\;R}\;+\;Time_{Frd}\;+\;Time_{Rcv}\;+\;Time_{Sign\;R}\; \end{array} \end{array}$$

The delay on the key sharing server is then taken into account by $Time_{Key\;Center}$ (Equation (10)). In this study, the procedure is referred to as $Time_{Vfy}$ and $Time_{Respns}$. The focus of this work is not on the key sharing server.

(10) $$\begin{array}{c} \begin{array}{c} Time_{Key\;Center}\;=Time_{Vfy}\;+\;Time_{Respns} \end{array} \end{array}$$

$Time_{Netork}$ is (Equation (11)) the delay on the divergent system, that consists of delay on the DSRC network between vehicles and RSUs ($Time_{Vehi\;-\;RSU}$), delay on the Web between RSUs and key sharing servers ($Time_{RSU\;-\;PKI}$), and random delay $▵Time_{Congeson}$ on the link between RSUs and key sharing center (which is ignored in [9]).

(11) $$\begin{array}{c} \begin{array}{c} Time_{Netork}\;=2\left(Time_{Vehi\;-\;RSU}\;+\;Time_{RSU\;-\;PKI}\right)\;+\;▵Time_{Congeson} \end{array} \end{array}$$

The delay is always avoided since the connections between the RSUs and cars are performed using the DSRC specifications [9]. The link amongst the RSUs and the key sharing server, on the other hand, is dependent on current web protocols, which, like some other web applications, may suffer from delay [44]. This Web delay ($▵Time_{Congeson}$) is modelled as an arbitrary delay that can happen over a time span and minimized as an extra communication time (0 to 30% extra communication time) in this simulation.

Then, as indicated in Section 3, a positive assumption’s ratio of β (0<β<1) is used to determine the expected key sharing delay. As a result, once the vehicle reaches an area with predetermined keys, the transmission delay will be $▵Time_{Switch\;Key}$, which is generated by network latency and is expected to be extremely minimal. Only when the assumptions fail will key requests be made, and the system shown in Figure 12 will be used.

Equation (12) should be used to estimate the overall delay of area-based key sharing, whereas $\sum Time_{Request\;Key}$ equals the sum of Equations (8)–(11).

(12) $$\begin{array}{c} \begin{array}{c} E\left(Time_{Delay}\right)=\beta .▵Time_{Switch\;Key}\;+\left(1-\beta \right).\sum Time_{Request\;Key} \end{array} \end{array}$$

The Equation (12), once β=0, which indicates that in this simulation, everything is the same. Vehicles must send key upgrading requests to the RSU, which is a requirement. As long as this method presume the condition in [9], it should be the same. Each vehicle will remain in a single zone for the duration of an AT (for example, 10 min).

Another significant point to note is that the [9] study did not account for speeding up the safety procedures such as information signing and verification on both cars and RSUs. The average time spent signing and validating on a vehicle ($Time_{Sign\;Vehi}+Time_{Vfy\;Vehi}$ in Equation (8)) is 50 ms, whereas the average time spent signing and confirming on RSUs ($Time_{Sign\;R}+Time_{Vfy\;R}$ in Equation (9)) is 200 ms, as per the findings presented in [9]. However, as demonstrated in Section 4, on vehicles that are fitted with acceleration, such as Nvidia Tegra systems, this average time required by safety computations can be reduced. This section of the approach simulates circumstances in which both automobiles and RSUs are fitted with Nvidia Tegra platforms, but neither vehicle nor RSU has such acceleration.

In the simulation, this method set it up so that at least one vehicle reaches an area per short time interval, and this procedure keep updating the transmission key. Some of them have the requisite keys, so the only key swap is necessary, while others do not and attempt to obtain a replacement key through the RSU. The successful assumptions ratios have been set at 0.3, 0.5 and 0.7.

**Figure 13:**
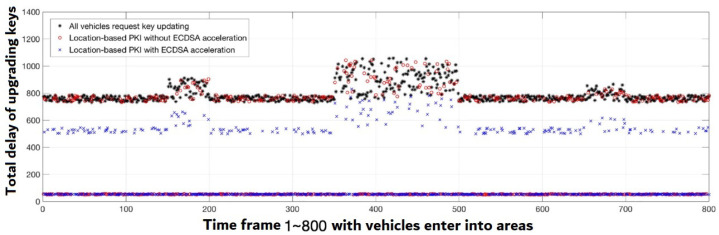
The findings on the Nvidia Tegra platform are compared to the different hardware implementation results based on the estimated time in [9].

Figure 13 depicts the delay sharing for the value of β=0.7. The findings on the Nvidia Tegra platform are compared to the different hardware implementation results based on the estimated time in [9]. It has been discovered that once the β is set to 0.7, which indicates a good accurate ratio of assumptions, most cars do not require upgrading key requests. The latency is equal to the time it takes to switch keys ($▵Time_{Swithc\;Key}$).

Furthermore, when the number of upgrading requests decreases, the average delay for key sharing decreases. Requests are made and executed in 2nd stage trust for some cars that do not have previously-shared keys for this area, as illustrated in Figure 12. Under this scenario, the Nvidia Tegra platform’s speeding up can minimize the ECDSA signature and authentication time, lowering key sharing delay.

**Figure 14:**
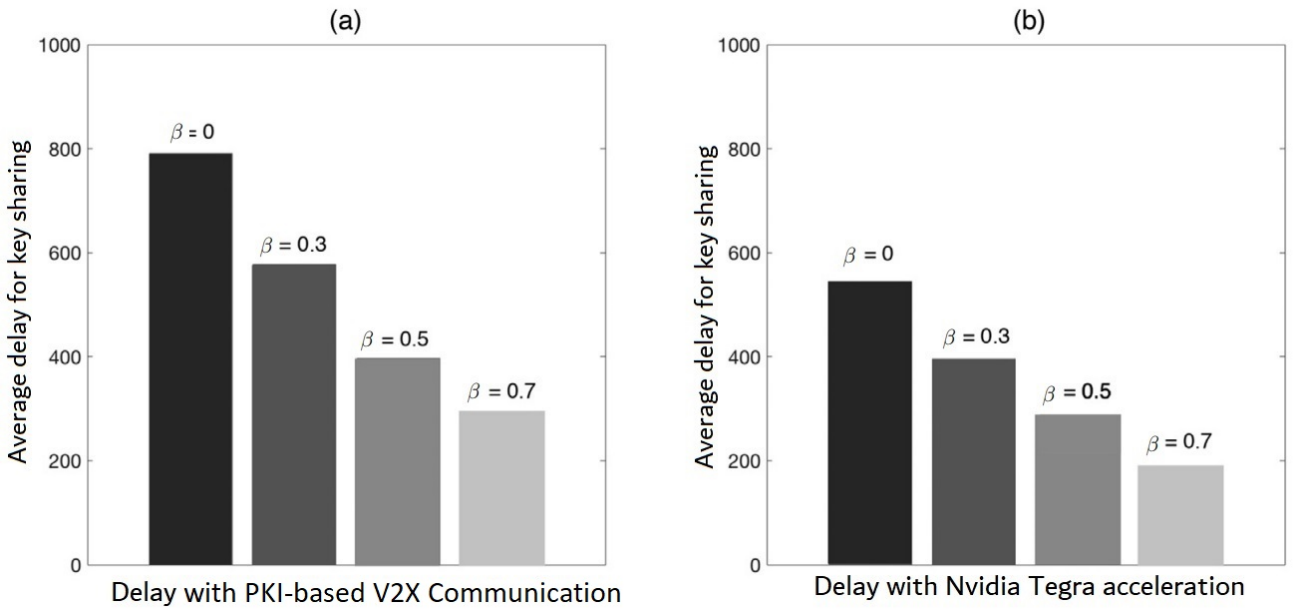
Comparisons of average delay caused by key sharing.

Figure 14 depicts the average delay caused by key sharing. Lower key sharing latency can be obtained with a greater accurate assumption ratio. The speeding up made by the introduction of the Nvidia Tegra SoC platform may be seen for the same assumption ratio.

This paper demonstrates the benefits of adopting an area-based key previously-sharing scheme in this simulation. First, by lowering request quantities, the area-based key sharing network can minimize the average delay. The average delay can be reduced with a high accuracy prediction ratio.

The second issue to consider is that key sharing is a form of information interchange process that includes data communication as well as the essential safety computations, whereas key requests/responses are a type of information conveyed in V2X divergent system. The time spent on safety computation activities is decreased by GPU acceleration, as per the assessed findings given in Figure 14. As a result, information interchange latency between cars and RSUs in V2X systems is significantly decreased for critical request/response.

For an input vector, the TLTPM system was constructed.

**Figure 15:**
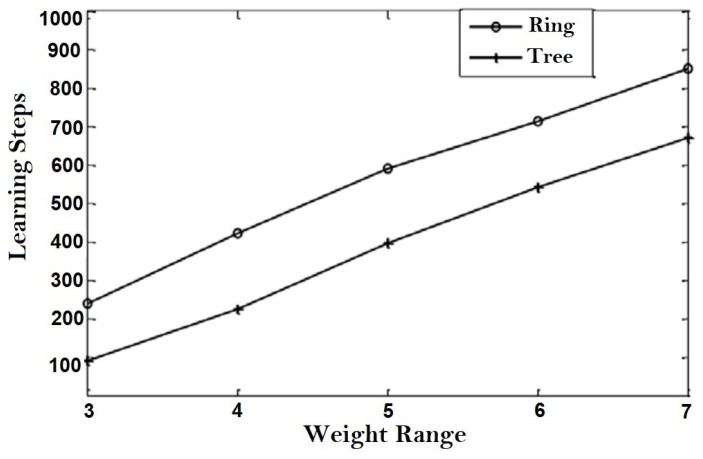
Average coordination stages for varying.

TLTPM syncing was first accomplished by utilizing optimum weights ranging from 3 to 7. Figure 15 shows the average number of learning phases necessary to reach weight coordination for various weight range values for both ring and tree topologies for a constant number of members m=8.

**Figure 16:**
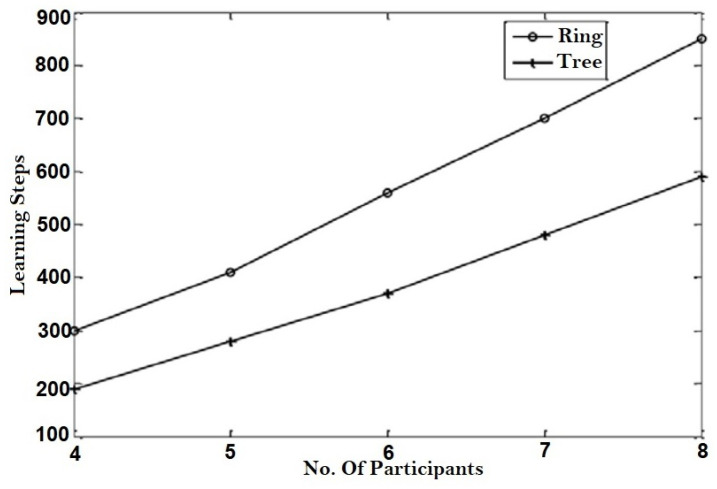
Average coordination stages for fixed weight range.

For such constant λ value of 7, Figure 16 displays the average range of learning stages plotted against the amount of users. As predicted, the coordination time increases as λ and the number of members increases. When contrasted to the ring system, the number of training necessary to generate a key utilizing tree structure is clearly fewer.

The following are the common problems to consider while designing group standards:

The proficiency of a group key exchange method may vary as the amount of the group of members varies. The method’s extensibility indicates to its capacity to be expanded to consider a broad group. Extensibility in the terms of confidentiality relates to the distribution and management of keys, as well as a variety of other safety regulations, when the group grows or shrinks. The group neural key created at the start of the connection can be used all across the session when interacting between fixed groups. However, in dynamic groups, where users enter and exit the group on a regular basis, the group key produced initially will not enough. This is to enable both reverse access control, which prevents a fresh member from capturing prior messages, and controlling of forward access, which prevents a departing user from viewing forthcoming communications. When a fresh member enters or an old member quits, the group key must be produced again. Individual Rekeying is the name given to this technique. As a result, group key configurations for strong communication groupings should allow users to join or immediately leave without jeopardizing future confidentiality, past confidentiality, or key autonomy.

The joining principle cannot obtain any of the previous keys since the “join” procedure must ensure full past confidentiality. As a result, each key must be changed. The “join” step in the suggested ring structure procedure results in the recreation of a fresh neural key by restarting the TLTPM coordination. If the new member joins as a current node, it combines with its adjoining and establishes a new set of coordinated weights before moving onto another stage; if it enters as a new user, this will possess its optimal weight vector to the final stage and prepare its system with its adjacent cluster for weight coordination.

Comparably, the “leave” procedure must ensure flawless forward confidentiality, then all the keys that perhaps the exiting member learns must be changed, that is accomplished in the ring structure procedure by bringing back the procedure each time a principal is inserted or deleted, while in the tree-like framework, if some member quits, the member who is linked to the former member, say $\left(USER\;5\right)$, will produce new weights and link with the adjacent group $\left(USER\;78\right)$. The procedure is repeated until the m−1 level is reached. As a result, the vital freshness and independence are retained.

**Figure 17:**
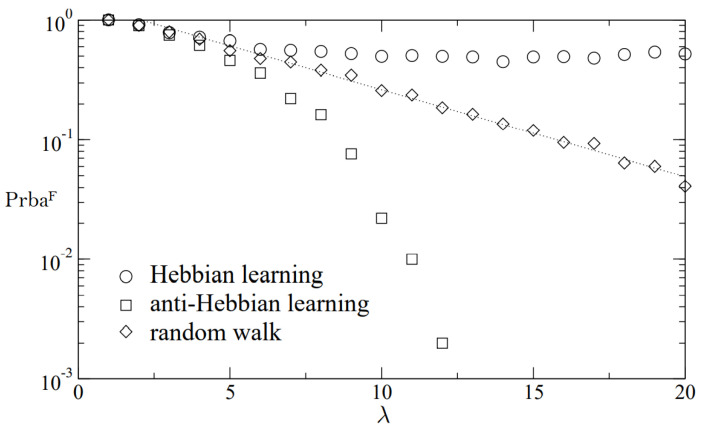
Possibility of a majority invasion with 100 assailant systems prevailing.

Figure 17 illustrates the probability of a majority approach with 100 attacker networks succeeding. In this case, 10,000 iterations are taken account and N=1000,$\tau_{1}=\tau_{2}\;=\tau_{3}=3$.

The findings of the NIST test [46] on the synced neuronal key are shown in Table 2. The *p*-Value evaluation is carried out for the TLTPM, CVTPM [10], Dolecki and Kozera [14], Liu et al. [34], VVTPM [12], and GAN-DLTPM [11] techniques. The TLTPM’s *p*-Value has indeed been emerged as a promising alternative than others.

The outcome of the frequency test in the synced neural key indicates a percentage of 1 and 0. The result is 0.701496, which is a lot better than the result 0.512374 in [10], 0.537924 in [14], 0.516357 in [34], 0.538632 in [12], 0.584326 in [11], 0.1329 in [35], 0.632558 in [36], and 0.629806 in [37]. Table 3 shows the *p*-Value comparisons for the frequency strategy.

Table 4 shows the outcomes for different τ1−τ2−τ3−N−λ values. Two columns define the minimum and maximum syncing times. The capacity of an adversary to formulate and implement the actions of approved TLTPMs is evaluated by invader syncing.

The arrangements (8-8-8-16-8) and (8-8-8-8-128) provide the optimum protection against the invading system, as shown in Table 4. In all 700,000 trials, E will not respond like A and B’s TLTPM. Table 5 displays the most cost-effective choices.

For various learning, weight range, and constant TLTPM size, Table 6 analyzes the synchronization periods of TLTPM and TPM-FPGA methods. When λ increases in all three rules, there is a propensity for the sync step to rise. In the λ range of 5 to 15, Hebbian hardly requires any sync stages than that of the other two rules, but when the λ rises, Hebbian uses more sync steps than the others.

The Anti-Hebbian harmonizing times of the TLTPM and CVTPM strategies are compared in Table 7. The TLTPM’s sync interval is noticeably shorter than the CVTPM’s, as shown in table. The Anti-Hebbian, in comparing to others, requires less effort.

**Figure 18:**
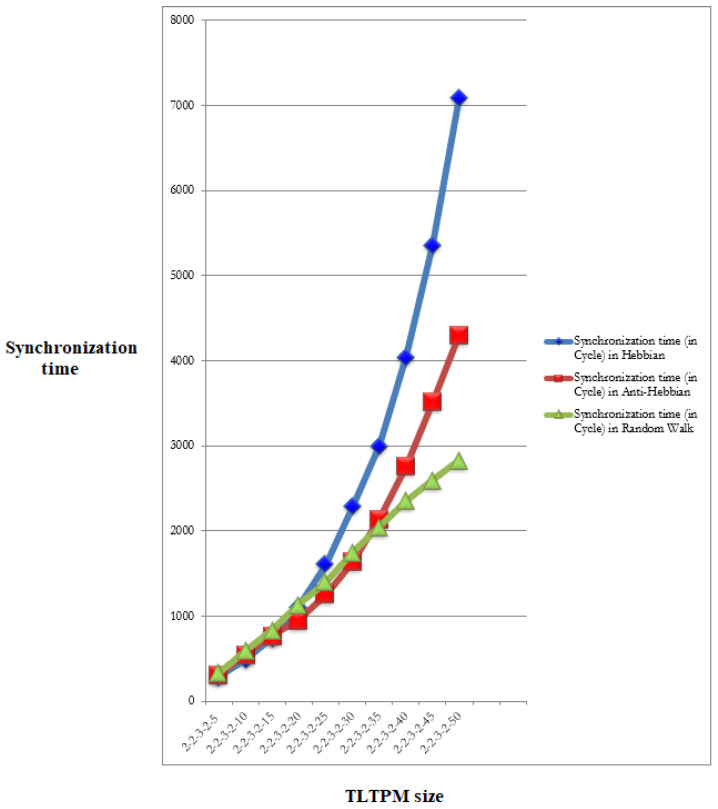
TLTPM size is fixed and 512-bit keys are generated with a varied weight range.

The necessary synchronizing duration for the 512-bit key generation with varying synaptic ranges is shown in Figure 18. In large scale networks, the Random Walk rule surpasses other learning techniques.

Table 8 compares the TLTPM and VVTPM time synchronization approaches using the Random Walk method. The TLTPM, which uses the Random Walk learning algorithm, has a much faster coordinating period than the VVTPM.

## 5. Conclusions and Future Scope

This paper introduced a data amalgamation trust mechanism for V2X divergent networks. To begin, a definition with four degrees of information amalgamation trust is provided based on V2X application requirements. Then, in order to successfully execute keys that serve as a foundation for all levels of information trust services in V2X networks, an area-based key sharing technique is created. Security-related calculations for data sharing, such as authenticated key exchange and extra data transmission, are taken into consideration and accelerated using GPGPU. It has been found that this trust mechanism significantly enhanced the quality of the current V2X network solution. The key sharing procedure may be viewed as one of the information communication processes that require the maximum level of confidence. This paper suggested that to establish the security framework by distributing security keys, a key sharing network, such as a PKI, is necessary. First, the area-based key distribution technique can reduce mean latency by decreasing request quantities. With a high accuracy prediction rate, the mean delay may be decreased. Second, key distribution is a type of information exchange method that comprises data transmission as well as key requests/responses, whereas key requests/responses are a data type that is transmitted in V2X heterogeneous networks. Third, GPU acceleration cuts down on the time it takes to perform security-related computations. As a result, the time it takes for essential requests and responses to be exchanged in V2X networks including cars and RSUs is greatly reduced. However, unlike other approaches to traffic assumptions, the assumptions in this study is based on the perspective of a single vehicle rather than anticipating traffic flows for entire region. To put it another way, the prediction is based on the historical route information of a single vehicle. This paper also includes a discussion among the most significant key switch over methods. This analysis looks at their flaws along with providing TLTPM group syncing. A TLTPM-based key interchange method could result in quicker key interchange network. TLTPM can increase safety in real-world scenarios by modifying the vector size, as shown in this research. The effectiveness of the approach, along with current TPM-FPGA, CVTPM, and VVTPM procedures, is assessed. Furthermore, simulations are conducted to find the best τ1−τ2−τ3−N and λ values for protecting against an intruding system. In varied learning rules, the TLTPM’s syncing duration are quicker than the existing TPM-FPGA, CVTPM, and VVTPM methods. The proposed technique’s shortcoming is that it does not use a deep learning algorithm to install vehicle route assumptions procedures in each vehicle end. It is instead deployed on the MITS central server. The average delay for key sharing, as indicated in Section 5, is largely dependent on the perfection of each vehicle’s path assumptions. However, for performance reasons, this method of assumptions should be installed on each vehicle end instead of the MITS’s central server. In the future, a deep learning technique can be used to deliver this type of assumption. In addition, we intend to provide a more generic data communication trust model for information amalgamation in V2X that includes key sharing.

## Figures and Tables

**Table 1:** The Symbols’ Meaning.

Symbol	Meaning
*N*	The quantity of input nodes that must be accessible to every node from the first concealed layer.
λ	Weight range
τ1	1st concealed layer node count
τ2	2nd concealed layer node count
τ3	3rd concealed layer node count
δ	Weight vector
γ	The input of neural system
ς	Number of input vectors
μ	Vector dimension
ξ	Output of hidden layer
κ	Computation result of hidden layer
ζ	Output of TLTPM
Φ	Heaviside stage function

**Table 2:** *p*-Value Comparisons.

Test	TLTPM’s *p*-Value	GAN-DLTPM’s *p*-Value [11]	VVTPM’s *p*-Value [12]	CVTPM’s *p*-Value [10]	*p*-Value of Liu et al. [34]	*p*-Value of Dolecki et al. [14]
Frequency	0.701496	0.584326	0.538632	0.512374	0.516357	0.537924
Frequency within a Block	0.636638	0.579271	0.551571	0.538721	0.541873	0.551972
Runs	0.584925	0.541851	0.508936	0.489451	0.481406	0.487329
Longest Run of Ones in a Block	0.12891	0.097367	0.061574	0.042081	0.047235	0.061287
Binary Matrix Rank	0.768372	0.689210	0.420589	0.417393	0.427351	0.473641
Discrete Fourier Transform	0.625826	0.412382	0.427985	0.392762	0.397219	0.417235
Non-overlapping Template Matching	0.556917	0.476302	0.463462	0.431759	0.447348	0.461319
Overlapping (Periodic) Template Matching	0.392084	0.219634	0.204875	0.170426	0.207321	0.181369
Maurer’s “Universal Statistical”	0.869737	0.786158	0.728347	0.689478	0.719872	0.701985
Linear Complexity	0.780859	0.655287	0.657894	0.594917	0.657410	0.631207
Serial	0.672865	0.520863	0.547216	0.478902	0.482737	0.531370
Approximate Entropy	0.413883	0.285963	0.280699	0.204175	0.287319	0.274696
Cummulative Sums	0.766967	0.693105	0.640273	0.587290	0.631971	0.643780
Random Excursions	0.526641	0.349342	0.377634	0.299875	0.361925	0.357354
Random Excursions Variants	0.442695	0.223387	0.249632	0.180542	0.247329	0.234691

**Table 3:** Frequency Test’s *p*-Value.

Methods	*p*-Value
TLTPM	0.701496
CVTPM [10]	0.512374
Dolecki and Kozera [14]	0.537924
Liu et al. [34]	0.516357
VVTPM [12]	0.538632
GAN-DLTPM [11]	0.584326
Karakaya et al. [35]	0.1329
Patidar et al. [36]	0.632558
Liu et al. [37]	0.629806

**Table 4:** Outcomes of 700,000 Iterations of Various τ1−τ2−τ3−N−λ.

τ1−τ2−τ3−N−λ	No. of Min Coordination Steps	No. of Max Coordination Steps	Min Coordination Time (sec.)	Max Coordination Time (sec.)	Attacker’s Successfull Syncing	% of Attacker’s Successfull Syncing
(1-1-1-512-1)	12	33	0.0616	3.3241	336,237	48.02
(2-2-2-256-1)	11	30	0.0697	2.2658	332,675	47.54
(4-4-4-128-1)	11	38	0.0773	2.2549	331,878	47.42
(8-8-8-64-1)	10	29	0.0725	3.0376	327,366	46.74
(1-1-1-256-2)	11	42	0.0699	3.1590	312,752	44.68
(2-2-2-128-2)	12	33	0.0681	652.7532	318,530	45.6
(4-4-4-64-2)	12	216	0.0655	112.2364	279,659	39.94
(8-8-8-32-2)	13	933	0.0588	69.2487	95,873	13.68
(1-1-1-128-8)	18	75	0.0617	2.0964	98,618	14.09
(2-2-2-64-8)	22	448	0.0777	3.8630	31,477	4.47
(4-4-4-32-8)	24	811	0.0496	6.5721	114	0.02
(8-8-8-16-8)	43	714	0.0335	0.0592	0	0.0
(1-1-1-64-128)	10	133	0.0218	0.6478	115,738	16.52
(2-2-2-32-128)	18	397	0.0546	3.4973	33,126	4.74
(4-4-4-16-128)	23	916	0.0379	4.0366	411	0.06
(8-8-8-8-128)	36	1118	0.0487	5.1897	0	0.0

**Table 5:** Outcomes of 1,000,000 iterations for the (8-8-8-16-8) and (8-8-8-8-128) set up.

τ1−τ2−τ3−N−λ	No. of Min Coordination Steps	No. of Max Coordination Steps	Min Coordination Time (sec.)	Max Coordination Time (sec.)	Attacker’s Successfull Syncing	% of Attacker’s Successfull Syncing
(8-8-8-16-8)	46	863	0.0839	2.8185	0	0
(8-8-8-8-128)	37	937	0.0417	1.3896	2	0.0002

**Table 6:** TLTPM and TPM-FPGA sync times are compared.

λ Value	TLTPM’s Coordination Period in Hebbian (Cycle)	TPM-FPGA’s Syncing Period in Hebbian (Cycle) [13]
5	31,256	34,064
10	68,134	71,038
15	99,736	130,827
20	237,463	267,253
25	351,209	398,702
30	499,846	537,261
35	698,327	742,894
40	831,203	896,735
45	1,101,629	1,173,418
50	1,443,959	1,498,329

**Table 7:** TLTPM and CVTPM syncing times are compared.

λ Value	TLTPM’s Coordination Period in Anti-Hebbian (Cycle)	CVTPM’s Syncing Period in Anti-Hebbian (Cycle) [10]
5	38,589	38,926
10	73,934	75,309
15	108,566	119,847
20	162,314	185,724
25	239,347	267,117
30	315,258	358,275
35	441,273	479,362
40	572,834	598,143
45	669,008	813,839
50	872,538	945,257

**Table 8:** A comparison of TLTPM and VVTPM sync times.

λ Value	TLTPM’s Coordination Period in Random Walk (Cycle)	VVTPM’s Syncing Period in Random Walk (Cycle) [12]
5	39,413	42,698
10	69,576	73,454
15	101,734	116,324
20	164,973	179,317
25	248,346	258,502
30	328,305	337,816
35	412,737	485,423
40	467,376	538,681
45	639,206	738,908
50	778,545	878,419

## Data Availability

The study did not report any data.
